# Effects of infliximab on lung and circulating natural killer cells, CD56+ T cells and B cells in sarcoidosis

**DOI:** 10.1136/bmjresp-2021-000933

**Published:** 2021-07-07

**Authors:** Susanna Kullberg, Natalia V Rivera, Johan Grunewald, Anders Eklund

**Affiliations:** 1 Department of Respiratory Medicine, Theme Inflammation and Ageing, Karolinska University Hospital, Stockholm, Sweden; 2 Respiratory Medicine Division, Department of Medicine, Karolinska Institute, Stockholm, Sweden

**Keywords:** lymphocyte biology, sarcoidosis, systemic disease and lungs

## Abstract

**Background:**

Tumour necrosis factor α (TNF-α) is pivotal in sarcoid granuloma formation, and inhibitors of TNF-α offer an attractive third-line treatment option in sarcoidosis. The sarcoid inflammation is characterised by an exaggerated T helper 1 response, and evidence indicates a contribution of dysregulated and/or deficient NK (natural killer) cells, CD56+ T cells and B cells.

**Objectives:**

Insight into how TNF-α inhibitors influence these cells may provide more information on inflammatory mechanisms in sarcoidosis and improve understanding of such treatment. We therefore evaluated treatment effects of the TNF-α inhibitor infliximab on lung and peripheral blood (PB) NK, CD56+ T cells and B cells.

**Methods:**

Fifteen patients were assessed with PB samples, spirometry and CT scan, and 11 of them also underwent bronchoalveolar lavage (BAL) close to start of infliximab treatment. These investigations were repeated after 6 months of treatment.

**Results:**

Twelve out of 15 patients disclosed a clinical improvement at follow-up. Median percentage of BAL fluid (BALF) CD56+ T cells increased while a decrease was seen in PB (p<0.05 and 0.005, respectively). No significant changes were observed for NK cells. There was a trend towards increased median percentage of PB B cells (p=0.07), and a negative correlation was observed between PB and BALF B cells after treatment (p<0.05).

**Conclusion:**

In conclusion, 6 months of infliximab treatment in patients with sarcoidosis, of whom the majority benefited from the treatment, influenced immune cells in the lung and circulation differently, highlighting the importance of investigating several compartments concomitantly when evaluating treatment effects on the inflammatory activity.

**Key messages**

- Can influence on natural killer cells, CD56+ T cells and B cells in the lung and circulation explain effects from treatment with tumour necrosis factor α (TNF-α) inhibitors in patients with sarcoidosis?
- Six months of treatment with the TNF-α inhibitor infliximab in patients with sarcoidosis, of whom the majority benefited from treatment, led to an increased median percentage of lung CD56+ T cells while a decrease was seen in the circulation.
- The results suggest different effects from infliximab treatment on lung and peripheral blood CD56+ T cells, respectively, focusing attention on the importance of investigating several compartments for an improved understanding of the inflammation in sarcoidosis.

## Introduction

Sarcoidosis is a systemic inflammatory disorder that predominantly involves the lungs and/or intrathoracic lymph nodes. The disease can be self-limiting, seen mostly in patients with the clinical phenotype Löfgren’s syndrome characterised by having an acute onset. However, the majority of patients, non-Löfgrens, usually will have an insidious onset and run a protracted disease course with more organ dysfunction.

In the lungs of affected individuals, a T helper 1 (TH1) alveolitis is seen with an accumulation of CD4+ T cells, elevated CD4/CD8 ratio, and release of proinflammatory cytokines, for example, tumour necrosis factor α (TNF-α) and interferon γ (IFN-γ).^1^ Eventually, non-necrotising granulomas are formed, which can lead to organ function impairment and sometimes failure.^1^

TNF-α inhibitors are used as a third-line treatment option for sarcoidosis. However, about 20% do not seem to benefit from treatment with TNF-α inhibitors. Also, at least half of the patients relapse after treatment discontinuation, and the optimal treatment duration and dose are not established.^2–4^

We have previously reported that 6 months of therapy with the TNF-α inhibitor infliximab led to a decreased percentage of bronchoalveolar lavage fluid (BALF) CD4+ T cells expressing the activation marker CD69 and a reduced CD4/CD8 ratio in patients classified as responders, indicating a less intensive TH1 alveolitis.^5^ To further explore the immunological mechanisms behind the effects of infliximab treatment, we set out to investigate its effect on natural killer (NK) cells, CD56+ T cells and B cells.

NK cells are lymphocytes expressing CD56 but not CD3 (CD3−CD56+). In BALF from patients with sarcoidosis, an increased frequency of a special phenotype of NK cells (CD56^bright^) with the capacity to produce high levels of TNF-α and IFN-γ was found, compared with BALF from healthy controls, and thereby has the potential to contribute to the TH1 alveolitis.^6^ Furthermore, it has been hypothesised that the release of IFN-γ from NK cells may stimulate TNF-α secretion from macrophages,^7^ further augmenting the TH1 response. An increased percentage of NK cells in BALF of patients with sarcoidosis is linked with a poor outcome and anticipates a need for steroid treatment.^8^

CD56 can also be expressed on CD3+ cells and can be found on a number of T cell subsets (eg, CD4+ and CD8+ T cells) and their functional divergence has been suggested to be dependent on the T cell subset and microenvironment.^9^ In peripheral blood (PB), most CD56+ T cells express CD8 and seem more efficient in IFN-γ production than CD56− T cells.^10^ Expansion of these cells has been described in conditions involving chronic activation of the immune system such as rheumatic and autoimmune diseases.^11^ In sarcoidosis, an increased number of CD56+ T cells has been found in PB compared with controls, while no difference was observed in BALF.^6 12^

Despite sarcoidosis being mainly considered a T cell-driven disease, several observations propose a B cell involvement and networking between these two cell types. An increased production of IgG and IgM is found in the sarcoid lung, and there is an association with polyclonal hypergammaglobulinaemia,^13^ and some patients seem to benefit from treatment with rituximab, a B cell inhibitor.^14^ B cell activating factor (BAFF) is vital for the maintenance and survival of B cells. In sarcoidosis, a positive correlation between disease activity and serum and BALF BAFF levels has been reported.^15 16^ A link with the TH1 alveolitis is also suggested by the finding that BAFF does not only stimulate B cells but also T cells through BAFF receptors and can augment TH1-associated inflammatory responses.^17 18^

How infliximab can influence NK cells, CD56+ T cells and B cells in sarcoidosis is not yet detangled and there are only scarce data from other inflammatory diseases.

The current study was designed to measure the therapeutic effects of infliximab on the expression of NK cells, CD56+ T cells, and B cells in the lung and circulation in patients with sarcoidosis by performing bronchoscopy with BAL and collecting blood samples close adjacent to the start of treatment and after 6 months of treatment.

## Methods

### Study design

Participants were identified among patients referred to the Department of Respiratory Medicine, Karolinska University Hospital, Stockholm, Sweden, with deteriorating sarcoidosis despite previous treatment with corticosteroids and/or methotrexate.

Before starting therapy with infliximab, patients were characterised with chest X-ray (classified according to Scadding’s staging system), CT scan and spirometry.

Bronchoscopy with BAL was performed on average 6 weeks before (range 3–9 weeks) the start of therapy. PB was drawn about 60 min prior to bronchoscopy. All investigations were repeated at follow-up after the first half year on treatment.

In order to make the study population as homogeneous as possible, the intention was to give as similar concomitant treatment as possible to all patients, 5 mg prednisone, and not change the dose between first and second bronchoscopy.

### Patient and public involvement

Patients were not involved in the design of the study, setting the research questions or outcome measures. Eligible patients were involved in recruitment. They were informed about the study as well as alternative treatments by their treating physicians (SK, JG and AE) and took an active part in the decision whether they should start treatment with infliximab. The patients had the option to receive infliximab treatment without participating in the study. All patients were informed about their personal outcome, that is, results from lung function tests and CT scan. Patients who were interested in BALF data were also informed about those results. The results will be disseminated to wider patient communities at so-called ‘Information day about sarcoidosis for patients’, an event arranged by our unit. Patients will not be directly involved in disseminating the results to medical caregivers and scientific communities.

### BALF and PB

Bronchoscopy with BAL was performed as previously described.^19^ Centrifugation was used to separate BALF and PB mononuclear cells. The percentage of lymphocyte subtypes was measured by triple-laser, eight-colour flow cytometry using a FACS Fortessa X-20 (Becton-Dickinson) with software FacsDIVA. A kit from BD (product number 337166) with the following antibody panel was used: CD3-FITC, CD19-APC, CD16/56-PE. CD4+ and CD8+ T cells were identified using antibodies CD4-APC-H7 clone SK3 and CD8-AmCyan clone SK1 (BD Biosciences, California, USA).

For subtyping, the following gating strategy was used—B cells: CD3−CD19+, T cells: CD3+, NK cells: CD3−CD16+CD56+, CD56+ T cells: CD3+CD16+CD56+.

Lymphocytes were distinguished on the basis of forward and side scatters, and the percentage of NK cells, CD56+ T cells and B cells was scored by calculation of positive cells in each specific quadrant.

### Infliximab therapy

Infliximab (Remicade, Merck Sharpe & Dome AB (Sweden) or Inflectra, Pfizer AB (Sweden) was administered intravenously 3–5 mg/kg body weight every 4–8 weeks after an initial induction phase. During the study period, a consensus document on infliximab therapy was published,^4^ recommending a dose of 5 mg/kg body weight and infusion every fourth week after the initial induction phase. Patients included thereafter (n=11) were therefore treated according to these recommendations, that is, given a higher dose and more frequently than patients included at the beginning of the study, a mean cumulative dose of 36 mg infliximab/kg body weight, compared with 16 mg, at follow-up.

### Definition of response

Response was defined as previously described.^5^ In brief, CT scans were independently evaluated by a radiologist and one of the researchers in the study (SK). Response was defined as either improved radiographic changes compatible with sarcoidosis, or stable radiographic changes despite a reduction in concomitant immunosuppressant therapy, while non-response was defined by increased radiographic changes and/or a need to increase concomitant immunosuppressant therapy.

### Data analysis

Cell measurements and lung function data were analysed using the non-parametric test Wilcoxon rank, for comparisons between pretreatment and post-treatment values. P value significance was set at <0.05. Descriptive statistics were used for calculation of median values and 25th–75th percentile. Additionally, correlation analysis was conducted using Spearman’s rank correlation test. All analyses were performed using Jamovi V.1.1.9.0 software (https://www.jamovi.org). Graphs and tables were created with Microsoft Excel 2013 and Microsoft Word 2013, respectively.

## Results

### Study subjects

Fifteen patients were included in the study, and 11 of these were subject to bronchoscopy and had BALF data analysis (see table 1). They were all of Caucasian origin. None of the included patients had a history of serious infections (including tuberculosis and hepatitis), congestive heart failure or malignancy. All patients were diagnosed with sarcoidosis (non-Löfgren’s syndrome) according to criteria established by the World Association of Sarcoidosis and Other Granulomatous Disorders.^20^ For clinical characteristics, see table 1.

**Table 1:** Baseline characteristics when infliximab therapy was initiated

Patient	Sex	Smoking	Age	Duration	Scadding	EPM	Treatment indication	Treatment	BAL
1	M	No	53	20	IV	Peripheral lymph nodes	Pulmonary	P	x
2	M	No	40	5	I	Ocular	Fatigue, joint pain	P	x
3	M	No	46	3	II	0	Pulmonary	P	x
4	M	No	42	2	II	0	Pulmonary	P	0
5	F	No	42	12	IV	0	Pulmonary	P	0
6	F	No	55	3	I	Skin	Fatigue	P	x
7	M	No	44	9	III	Hypercalciuria	Pulmonary	P	x
8	M	No	44	4	II	0	Pulmonary	M	x
9	M	No	50	4	IV	0	Pulmonary	P	x
10	M	No	55	2	II	Skin	Pulmonary	P	x
11	M	No	51	6	IV	0	Pulmonary	P	x
12	F	Yes	42	2	II	0	Pulmonary	M	0
13	M	Yes	34	6	II	Peripheral lymph nodes	Pulmonary	M	0
14	M	No	51	7	IV	0	Pulmonary	P	x
15	M	Yes	49	4	II	0	Pulmonary	0	x

Baseline characteristics when infliximab therapy was initiated. Smoking=current smoking habits, no=not a current smoker, yes=current smoker; duration=duration of sarcoidosis in years before infliximab treatment started; treatment indication=which symptoms that were the reason for initiation of infliximab treatment; Scadding=Scadding stage=radiographic extent of sarcoidosis assessed by chest X-ray (0–IV); treatment=concomitant treatment during infliximab treatment. P, M and 0 denote prednisone, methotrexate and no concomitant treatment, respectively. The x and 0 in the BAL column represent if patient is included in BALF data analysis (x) or not (0).

BAL, bronchoalveolar lavage; BALF, BAL fluid; EPM, extrapulmonary manifestations; F, female; M, male.

All patients except number 2 had a history of both prednisone and methotrexate treatment. Patient number 2 was regarded as having a very active disease despite high-dose prednisone, and the clinical decision was that it was better for the patient to start with infliximab than methotrexate, which was also the wish of the patient. For those patients who did not tolerate a reduction of prednisone to 5 mg per day (patient numbers 2, 6 and 7), the dose was kept at a dose which relieved the symptoms best and the dose was then tapered down. Patient number 15 did not take any concomitant immunosuppressant at all during the study period due to misunderstanding. For various reasons, patient numbers 8, 12 and 13 received methotrexate as concomitant treatment. For detailed information on individual treatment, see online supplemental 1.

10.1136/bmjresp-2021-000933.supp1 Supplementary data

The majority of patients tolerated the treatment well without adverse events. Patient number 5 developed a possible adverse event with an increasing elevation of liver enzymes. The infliximab treatment was stopped after the fifth infusion, and the patient was excluded from the study with no participation in the follow-up. Patient number 4 became obstructive after the first bronchoscopy, recovery was bad and the BALF was not sufficient for analysis, therefore the procedure was not repeated. At the follow-up bronchoscopy, BALF from patient number 12, who was a smoker, was very grey and full of macrophages that lymphocyte data could not be retrieved with certainty, and therefore also this patient was excluded from BALF data analysis. Patient number 13 did not want to take part in the bronchoscopy part of the study.

Following response assessment, 12 patients were classified as responders and 2, both disclosing increasing sarcoidosis-related changes on CT scan, as non-responders. Both non-responders were found among the four patients administered with a lower mean cumulative dose of infliximab/kg body weight.

The two patients in Scadding stage I, with a non-pulmonary treatment indication (patient numbers 2 and 6), were classified as responders as the prednisone dose could be reduced after initiation of infliximab treatment. In addition, patient number 6 had parenchymal noduli, visible on the CT scan, and these were clearly reduced at follow-up.

### Lung function

Responders disclosed an increase in median percentage predicted forced vital capacity (FVC) from 71% (25th percentile 60–75th percentile 82) to 83% (71–94), and median percentage predicted forced expiratory volume in one second (FEV1) from 61% (41–75) to 67% (46–90) at follow-up (p<0.05 for both). Median percentage of diffusion capacity of the lung for carbon monoxide (DLCO) increased from 68% (62–81) to 78% (64–82), but this did not reach statistical significance. In the two non-responders, the median percentage of predicted FVC and FEV1 was more or less unchanged between baseline and follow-up, from 67% to 65% and 59% to 61%, respectively, while DLCO decreased from 57% to 48%.

### BALF and PB cells

**Figure 1:**
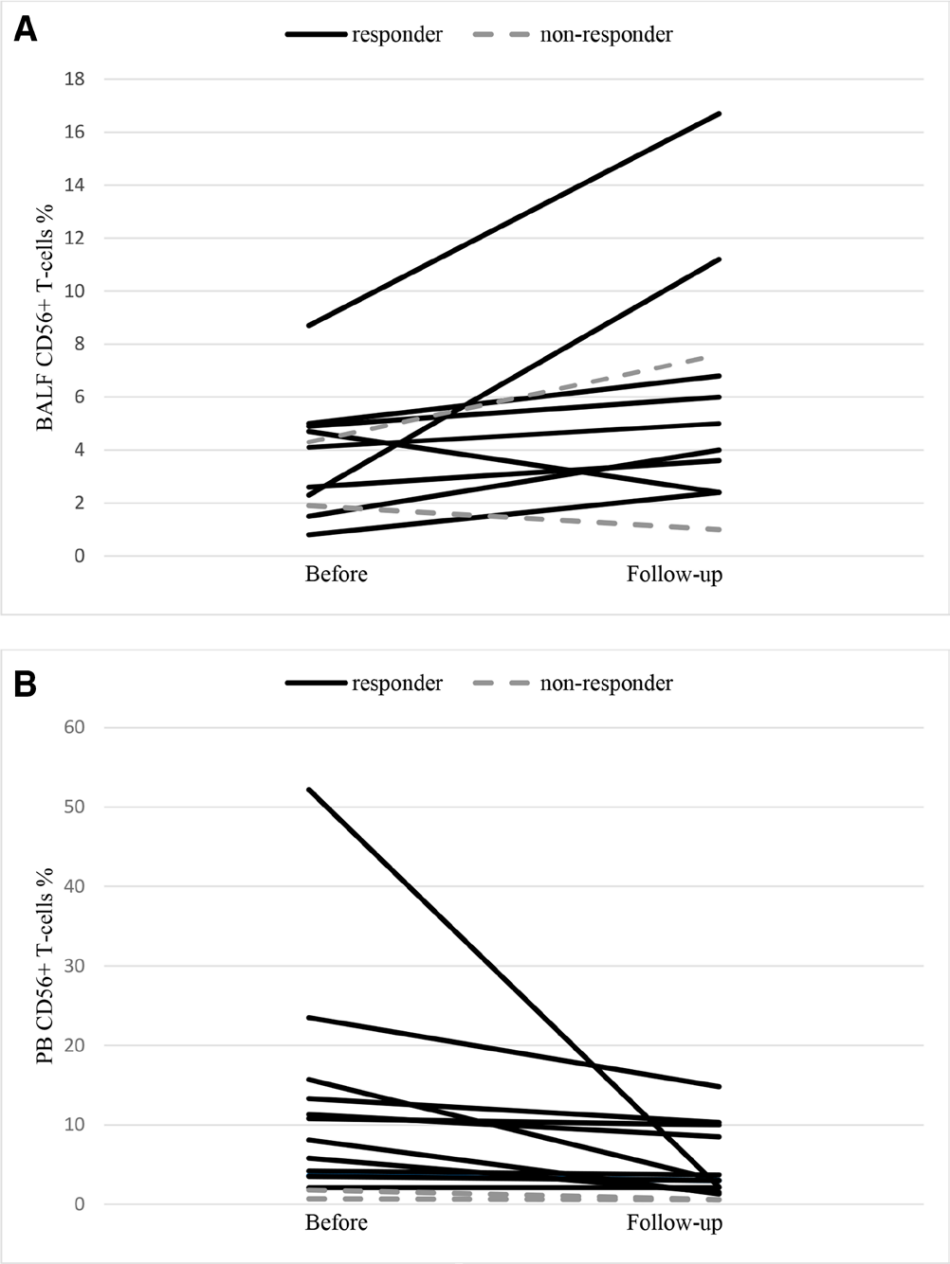
(A) Median percentage BALF CD56+ T cells before infliximab treatment and at follow-up in responders (n=9) and non-responders (n=2). Each line denotes one patient. (B) Median percentage PB CD56+ T cells before infliximab treatment and at follow-up in responders (n=12) and non-responders (n=2). Each line denotes one patient. BALF, bronchoalveolar lavage fluid; PB, peripheral blood.

After treatment, the median percentage of BALF CD56+ T cells increased significantly (from 4.1% (25th percentile 2.1–75th percentile 4.8) to 5.0% (3.0–5.2), p<0.05) while a reduction was seen in PB (from 7.0% (3.5–12.8) to 3.0% (1.7–7.3), p<0.005). However, no correlation was detected between BALF and PB CD56+ T cells, neither before nor after treatment (p>0.05). Individual data are shown in figure 1A, B.

No significant changes were seen in the median percentage of BALF and PB NK between baseline and follow-up (from 3.3% (1.9–6.6) to 1.8% (1.5–5.6) and from 17.0% (13.5–21.4) to 12.3% (9.1–15.3), respectively), p=0.2 for both. However, there was a variation between patients with both increased, decreased, and more or less unchanged median percentage values.

**Figure 2:**
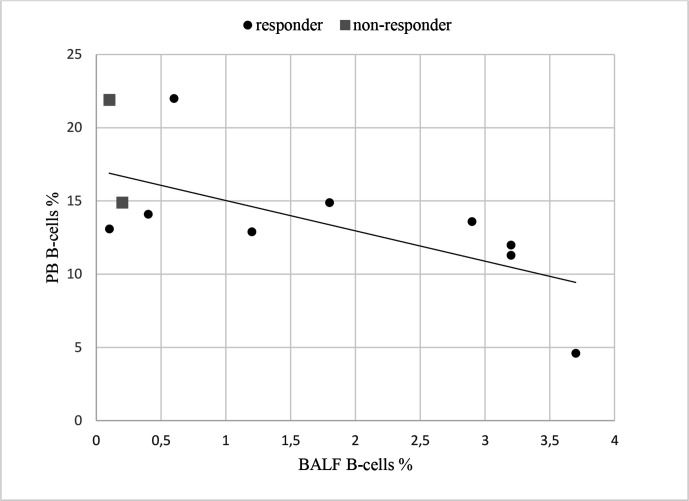
Correlation between percentage PB and BALF B cells after treatment in patients included in the bronchoscopy part (n=11). BALF, bronchoalveolar lavage fluid; PB, peripheral blood.

No significant change was seen in BALF B cells, median percentage decreased from 0.7% (0.3–0.9) to 0.6% (0.3–3.1) (p=0.4). In PB, B cells showed a tendency to increase after treatment, from 12.3% (9.6–13.5) to 13.8% (12.2–15.8) (p=0.07). After treatment, there was a negative correlation between PB and BALF B cells (Spearman’s r=−0.7, p<0.05) (see figure 2).

Detailed individual information on BALF and PB data is given in online supplemental 2A, B.

BALF data on cell concentration, percentages of macrophages and lymphocytes, as well as CD4/CD8 ratio for the majority of included patients have been published previously^5^ and can be found, including new information, in online supplemental 3.

## Discussion

We found that 6 months of infliximab treatment in patients with sarcoidosis refractory to conventional treatment led to an increased median percentage of BALF CD56+ T cells and a concomitant decrease in PB. The median percentage of PB B cells showed a tendency to increase, and a negative correlation between PB and BALF B cells was observed after treatment.

Patients with sarcoidosis often disclose a PB lymphopenia and research findings suggest that lymphocytes are depleted in PB due to increased infiltration in affected organs including the lung.^21 22^ Thus, it seems as if lung and PB lymphocytes can migrate between compartments, and an attractive explanation to the findings reported here on CD56+ T cells could be an influx of these cells from the circulation to the lung. But speaking against this theory does the fact that we did not detect a significant correlation between BALF and PB CD56+ T cells. Hence, the CD56+ T cells in BALF and PB might belong to different subpopulations. However, a lack of correlation can also be due to the limited number of included patients.

The practical bearing of CD56+ T cell expansion in PB from patients with sarcoidosis is unclear.^6 12^ However, given their potential of producing IFN-γ^9^ and our observation of a decrease paralleled by clinical improvement in most patients after infliximab therapy suggest that CD56+ T cells contribute to systemic inflammation. Likewise, in other inflammatory diseases, PB CD56+ T cells decreased after pharmacological intervention. Specifically, a study showed that treatment with infliximab restored PB CD56+ T cells to normal levels in patients with spondyloarthropathy and rheumatoid arthritis with high baseline values.^23^ In Behcet’s uveitis, proportions of IFN-γ-producing CD56+ T cells in PB were significantly higher than in healthy controls but normalised after treatment with prednisone and cyclophosphamide.^24^

In contrast to PB, the median percentage of CD56+ T cells in BALF increased. Since BALF CD56+ T cells are efficient producers of IFN-γ and TNF-α, even more efficient than PB CD56+ T cells following stimulation,^25^ we assumed that BALF CD56+ T cells would contribute to the TH1 alveolitis. However, our results point in the opposite direction; the increase in CD56+ T cells in BALF coincides with a decreased TH1 alveolitis.^5^ As the function of CD56+ T cells seems, at least to some degree, to be dependent on the T cell subset and microenvironment,^9^ we believe that the increased percentage of CD56+ T cells observed in BALF after treatment represents a CD56+ T cell population with a different function than in PB.

Interestingly, CD3 and NK cells-associated marker CD56/16 have been used in several studies on sarcoidosis and other lung diseases to identify NK T cells.^26–28^ NK T cells can release cytokines that can influence the balance between TH1 and TH2 responses.^29^ A dysregulation of NK T cells has been proposed to, at least partly, explain the amplified T cell response in sarcoidosis.^30^ Lower NK T cell frequencies in BALF have been correlated with exaggerated lymphocytosis and CD4+ T cell responses^31^ However, not all cells expressing these markers have the functional characteristics of NK T cells, and not all NK T cells express NK cell antigens.^29^ Still, it is possible that the BALF CD56+ T cells investigated here, at least to some extent, reflect NK T cells and thus, an expansion in BALF after infliximab treatment could explain the downregulated TH1 response. Moreover, supporting the hypothesis of BALF and PB CD56+ T cells having different functions is the finding that the majority of CD56+ T cells in PB do not express Vα24, a T cell receptor α-chain, which is regarded as a more accurate marker for identification of most NK T cells compared with CD56.^10 29^

Somewhat surprisingly, having in mind the capacity of NK cells to produce large amounts of IFN-γ and TNF-α,^6^ and as an increased percentage of NK cells in the BALF from patients with sarcoidosis associated with a poor outcome,^8^ we did not detect any significant changes between baseline and follow-up in NK cells. Also in RA, infliximab treatment led to decreased NK cell counts in PB, possibly through binding of membrane-associated TNF-α.^32^ However, we do not know if the NK cell function may be altered. Also, we observed individual differences, which may be of importance for outcome in the long run and risk of relapse.

Many studies have demonstrated signs of B cell activation in sarcoidosis.^16 33^ However, in PB of patients with serious chronic sarcoidosis, B cell lymphopenia as a result of memory B cells reduction was reported.^34^ Furthermore, patients with active chronic sarcoidosis had less circulating memory B cells compared with healthy controls and patients with inactive sarcoidosis.^15^ It has been suggested that the B cell lymphopenia is a consequence of these cells being attracted by cytokines to the lung where they are activated by T cells, subsequently differentiating into plasma cells, eventually leading to polyclonal hypergammaglobulinaemia.^13 34^

As we did not use markers for B cell subtypes, we cannot tell whether infliximab treatment had an impact on memory B cells in our patients with sarcoidosis. However, it is tempting to speculate that the tendency of increased median percentage of PB B cells after treatment might reflect a restoration of memory B cells by infliximab also in sarcoidosis. Since memory B cells can act as regulators of inflammation by producing suppressive cytokines,^35^ a memory B cell restoration could possibly contribute to the downregulated inflammation and clinical improvement. Moreover, if a powerful B cell response is necessary for a humoral response, a restoration of B cells may help to get rid of antigens that induce granuloma formation.^36^ Interestingly, in Crohn’s disease, another granulomatous disease, IgM memory B cell reduction in the circulation was restored after treatment with infliximab.^37^ Another study reported high serum BAFF concentrations in patients with Crohn’s disease responding to infliximab treatment compared with controls and non-responders, and this was reduced after treatment.^38^ Also, in spondyloarthropathy, infliximab treatment increased circulating memory B cells.^39^ In this context, we think our findings of a tendency to increased percentage of PB B cells and a significant negative correlation between PB and BALF B cells after, but not before treatment, may indicate that a disturbed B cell homeostasis, including dysregulated BAFF, is restored from infliximab. Notably, considering that BAFF can augment TH1 responses, control and reduction of BAFF expression have been suggested to provide a therapeutic strategy for sarcoidosis.^16^

This study has some major limitations. The lack of marker measurements for B and T cell subsets complicates the interpretation of functional consequences from the observed differences between compartments. The relatively small study sample with an imbalanced sex distribution, both smokers and non-smokers, and only two non-responders, limits us to draw firm conclusions on the observed changes concerning treatment outcome.

Major strengths include exploration of immune cells that have not been previously studied in relation to infliximab therapy in two compartments, that is, lung and circulation, before and after treatment.

## Conclusions

In conclusion, we observed a significant increase in the median percentage of BALF CD56+ T cells, while a significant decrease in PB in patients with sarcoidosis after 6 months of infliximab treatment. We found no significant changes for NK cells, though individual differences were observed. The median percentage of PB B cells showed a tendency to increase, and there was a negative correlation between PB and BALF B cells after treatment. The observed changes suggest different effects of infliximab treatment between compartments and highlight the importance of studying several compartments when investigating sarcoidosis.

It will be interesting to continue trying to detangle the effects of infliximab on immune cells and its relation to outcome in the long run. Nevertheless, we will continue our efforts to follow the patients longitudinally, collect more patient data, and look into more in-depth immune cells by broadening the antibody panel to B and T cell subsets, BAFF and BAFF receptors.

## Data Availability

All data relevant to the study are included in the article or uploaded as supplemental information. Data are available on reasonable request from the corresponding author.

## References

[1] Grunewald J, Grutters JC, Arkema EV, et al. Sarcoidosis. Nat Rev Dis Primers 2019;5:45. 10.1038/s41572-019-0096-x 31273209

[2] Adler BL, Wang CJ, Bui T-L, et al. Anti-Tumor necrosis factor agents in sarcoidosis: a systematic review of efficacy and safety. Semin Arthritis Rheum 2019;48:1093–104. 10.1016/j.semarthrit.2018.10.005 30446173

[3] Vorselaars ADM, Verwoerd A, van Moorsel CHM, et al. Prediction of relapse after discontinuation of infliximab therapy in severe sarcoidosis. Eur Respir J 2014;43:602–9. 10.1183/09031936.00055213 23988768

[4] Drent M, Cremers JP, Jansen TL, et al. Practical eminence and experience-based recommendations for use of TNF-α inhibitors in sarcoidosis. Sarcoidosis Vasc Diffuse Lung Dis 2014;31:91–107. 25078637

[5] Kullberg S, Rivera NV, Abo Al Hayja M, et al. Changes in lung immune cells related to clinical outcome during treatment with infliximab for sarcoidosis. Clin Exp Immunol 2020;201:85–93. 10.1111/cei.13438 32275772 PMC7290087

[6] Katchar K, Söderström K, Wahlstrom J, et al. Characterisation of natural killer cells and CD56+ T-cells in sarcoidosis patients. Eur Respir J 2005;26:77–85. 10.1183/09031936.05.00030805 15994392

[7] Ziegenhagen MW, Rothe ME, Zissel G, et al. Exaggerated TNFalpha release of alveolar macrophages in corticosteroid resistant sarcoidosis. Sarcoidosis Vasc Diffuse Lung Dis 2002;19:185–90. 12405487

[8] Tutor-Ureta P, Citores MJ, Castejón R, et al. Prognostic value of neutrophils and NK cells in bronchoalveolar lavage of sarcoidosis. Cytometry B Clin Cytom 2006;70:416–22. 10.1002/cyto.b.20120 16977633

[9] Van Acker HH, Capsomidis A, Smits EL, et al. Cd56 in the immune system: more than a marker for cytotoxicity? Front Immunol 2017;8:892. 10.3389/fimmu.2017.00892 28791027 PMC5522883

[10] Ohkawa T, Seki S, Dobashi H, et al. Systematic characterization of human CD8+ T cells with natural killer cell markers in comparison with natural killer cells and normal CD8+ T cells. Immunology 2001;103:281–90. 10.1046/j.1365-2567.2001.01248.x 11454057 PMC1783250

[11] Tarazona R, DelaRosa O, Alonso C, et al. Increased expression of NK cell markers on T lymphocytes in aging and chronic activation of the immune system reflects the accumulation of effector/senescent T cells. Mech Ageing Dev 2000;121:77–88. 10.1016/S0047-6374(00)00199-8 11164462

[12] Naccache J-M, Kambouchner M, Schischmanoff PO, et al. Increasing level of CD56+ T-cells in peripheral blood in sarcoidosis. Eur Respir J 2006;27:654. 10.1183/09031936.06.00129505 16507870

[13] Hunninghake GW, Crystal RG. Mechanisms of Hypergammaglobulinemia in pulmonary sarcoidosis. site of increased antibody production and role of T lymphocytes. J Clin Invest 1981;67:86–92. 10.1172/JCI110036 6969734 PMC371575

[14] Sweiss NJ, Lower EE, Mirsaeidi M, et al. Rituximab in the treatment of refractory pulmonary sarcoidosis. Eur Respir J 2014;43:1525–8. 10.1183/09031936.00224513 24488568 PMC4167390

[15] Saussine A, Tazi A, Feuillet S, et al. Active chronic sarcoidosis is characterized by increased transitional blood B cells, increased IL-10-producing regulatory B cells and high BAFF levels. PLoS One 2012;7:e43588. 10.1371/journal.pone.0043588 22927996 PMC3425471

[16] Ando M, Goto A, Takeno Y, et al. Significant elevation of the levels of B-cell activating factor (BAFF) in patients with sarcoidosis. Clin Rheumatol 2018;37:2833–8. 10.1007/s10067-018-4183-2 29936689

[17] Sutherland APR, Ng LG, Fletcher CA, et al. Baff augments certain Th1-associated inflammatory responses. J Immunol 2005;174:5537–44. 10.4049/jimmunol.174.9.5537 15843552

[18] Ng LG, Sutherland APR, Newton R, et al. B cell-activating factor belonging to the TNF family (BAFF)-R is the principal BAFF receptor facilitating BAFF costimulation of circulating T and B cells. J Immunol 2004;173:807–17. 10.4049/jimmunol.173.2.807 15240667

[19] Olsen HH, Grunewald J, Tornling G, et al. Bronchoalveolar lavage results are independent of season, age, gender and collection site. PLoS One 2012;7:e43644. 10.1371/journal.pone.0043644 22952729 PMC3432041

[20] Hunninghake GW, Costabel U, Ando M, et al. ATS/ERS/WASOG statement on sarcoidosis. American thoracic Society/European respiratory Society/World association of sarcoidosis and other granulomatous disorders. Sarcoidosis Vasc Diffuse Lung Dis 1999;16:149–73. 10560120

[21] Sweiss NJ, Salloum R, Gandhi S, et al. Significant CD4, CD8, and CD19 lymphopenia in peripheral blood of sarcoidosis patients correlates with severe disease manifestations. PLoS One 2010;5:e9088. 10.1371/journal.pone.0009088 20140091 PMC2816716

[22] Baughman RP, Hurtubise PE. Systemic immune response of patients with active pulmonary sarcoidosis. Clin Exp Immunol 1985;61:535–41. 2416496 PMC1577287

[23] Baeten D, Van Damme N, Van den Bosch F, et al. Impaired Th1 cytokine production in spondyloarthropathy is restored by anti-TNFalpha. Ann Rheum Dis 2001;60:750–5. 10.1136/ard.60.8.750 11454638 PMC1753790

[24] Ahn JK, Seo J-M, Yu J, et al. Down-Regulation of IFN-gamma-producing CD56+ T cells after combined low-dose cyclosporine/prednisone treatment in patients with Behçet's uveitis. Invest Ophthalmol Vis Sci 2005;46:2458–64. 10.1167/iovs.04-0792 15980236

[25] Katchar K, Wahlström J, Eklund A, et al. Highly activated T-cell receptor AV2S3(+) CD4(+) lung T-cell expansions in pulmonary sarcoidosis. Am J Respir Crit Care Med 2001;163:1540–5. 10.1164/ajrccm.163.7.2005028 11401870

[26] Sokhatska O, Padrão E, Sousa-Pinto B, et al. Nk and NKT cells in the diagnosis of diffuse lung diseases presenting with a lymphocytic alveolitis. BMC Pulm Med 2019;19:39. 10.1186/s12890-019-0802-1 30760244 PMC6373142

[27] Tøndell A, Rø AD, Åsberg A, et al. Activated CD8(+) T cells and NKT cells in BAL fluid improve diagnostic accuracy in sarcoidosis. Lung 2014;192:133–40. 10.1007/s00408-013-9527-8 24213536

[28] Bergantini L, Cameli P, d'Alessandro M, et al. Nk and NKT-like cells in granulomatous and fibrotic lung diseases. Clin Exp Med 2019;19:487–94. 10.1007/s10238-019-00578-3 31485847

[29] Berzins SP, Smyth MJ, Baxter AG. Presumed guilty: natural killer T cell defects and human disease. Nat Rev Immunol 2011;11:131–42. 10.1038/nri2904 21267014

[30] Grunewald J, Eklund A. Role of CD4+ T cells in sarcoidosis. Proc Am Thorac Soc 2007;4:461–4. 10.1513/pats.200606-130MS 17684290 PMC2647597

[31] Korosec P, Rijavec M, Silar M, et al. Deficiency of pulmonary Valpha24 Vbeta11 natural killer T cells in corticosteroid-naïve sarcoidosis patients. Respir Med 2010;104:571–7. 10.1016/j.rmed.2009.11.008 19954940

[32] Coulthard LR, Geiler J, Mathews RJ, et al. Differential effects of infliximab on absolute circulating blood leucocyte counts of innate immune cells in early and late rheumatoid arthritis patients. Clin Exp Immunol 2012;170:36–46. 10.1111/j.1365-2249.2012.04626.x 22943199 PMC3444715

[33] Kudryavtsev I, Serebriakova M, Starshinova A, et al. Imbalance in B cell and T follicular helper cell subsets in pulmonary sarcoidosis. Sci Rep 2020;10:1059. 10.1038/s41598-020-57741-0 31974463 PMC6978348

[34] Lee N-S, Barber L, Akula SM, et al. Disturbed homeostasis and multiple signaling defects in the peripheral blood B-cell compartment of patients with severe chronic sarcoidosis. Clin Vaccine Immunol 2011;18:1306–16. 10.1128/CVI.05118-11 21653741 PMC3147362

[35] Lund FE. Cytokine-Producing B lymphocytes-key regulators of immunity. Curr Opin Immunol 2008;20:332–8. 10.1016/j.coi.2008.03.003 18417336 PMC2474694

[36] Moller DR, Chen ES. Genetic basis of remitting sarcoidosis: triumph of the trimolecular complex? Am J Respir Cell Mol Biol 2002;27:391–5. 10.1165/rcmb.2002-0164PS 12356571

[37] Timmermans WMC, van Laar JAM, van der Houwen TB, et al. B-Cell dysregulation in Crohn's disease is partially restored with infliximab therapy. PLoS One 2016;11:e0160103. 10.1371/journal.pone.0160103 27468085 PMC4965034

[38] Andreou N-P, Legaki E, Dovrolis N, et al. B-Cell activating factor (BAFF) expression is associated with Crohn's disease and can serve as a potential prognostic indicator of disease response to infliximab treatment. Dig Liver Dis 2021;53:574-580. 10.1016/j.dld.2020.11.030 33339749

[39] Salinas GF, De Rycke L, Barendregt B, et al. Anti-Tnf treatment blocks the induction of T cell-dependent humoral responses. Ann Rheum Dis 2013;72:1037–43. 10.1136/annrheumdis-2011-201270 22968102

