# Supplementary material for: Effects of infliximab on lung and circulating natural killer cells, CD56+ T cells and B cells in sarcoidosis
Source: BMJ Open Respir Res. 2021 Jul 7;8(1):e000933. doi: 10.1136/bmjresp-2021-000933 (PMC8264913; doi:10.1136/bmjresp-2021-000933)
Supplement: Supplementary data [file bmjresp-2021-000933supp001.pdf]

**Patient number**	**Treatment at 1st bronchoscopy**	**Treatment at 2nd bronchoscopy/ follow-up**
1	5 mg prednisone	5 mg prednisone
2	20 mg prednisone	5 mg prednisone
3	0	5 mg prednisone
4	5 mg prednisone	5 mg prednisone
5	5 mg prednisone	nd
6	10 mg prednisone	10 mg prednisone
7	15 mg prednisone	5 mg prednisone
8	5 mg methotrexate	5 mg methotrexate
9	5 mg prednisone	5 mg prednisone
10	5 mg prednisone	5 mg prednisone
11	5 mg prednisone	5 mg prednisone
12	20 mg hydrocortisone<br>7.5 mg methotrexate	15 mg hydrocortisone<br>7.5 mg methotrexate
13	5 mg methotrexate	5 mg methotrexate
14	1.25 mg prednisone	1.25 mg prednisone
15	15 mg methotrexate	0

Supplement 1. Detailed information on immunosuppressant therapy in study subjects, nd= not determined. Patient number 3 was put on prednisone immediately after the 1st bronchoscopy. The prednisone dose inpatient number 6 was decreased directly after the 2nd bronchoscopy. Hydrocortisone treatment in patient number 12 was prescribed by a specialist in endocrinology for adrenal insufficiency, a consequence of previous prednisone treatment. Patient number 8 suffered from a psychiatric disease, which had deteriorated during prednisone treatment, and

Kullberg S, *et al. BMJ Open Resp Res* 2022; 8:e000933. doi: 10.1136/bmjresp-2021-000933

therefore, this patient was put on a low dose of methotrexate as concomitant treatment. Both patient number 12 and 13 experienced more side-effects from prednisone than methotrexate, and therefore also these 2 patients received methotrexate as concomitant treatment.

Kullberg S, *et al. BMJ Open Resp Res* 2022; 8:e000933. doi: 10.1136/bmjresp-2021-000933

Patient	NK cells		CD56+ T cells		B cells	
	Before	Follow-up	Before	Follow-up	Before	Follow-up
1	3.2	3.0	0.8	1.2	2.3	11.2
2	3.3	0.9	0.9	0.4	4.7	2.4
3	14.2	10.3	2.1	3.2	5.0	6.8
4	nd	nd	nd	nd	nd	nd
5	0.9	nd	0.2	nd	0.6	nd
6	1.6	1.6	0.9	3.7	0.8	2.4
7	5.8	6.9	0.6	2.9	1.5	5.0
8	4.5	1.4	0.3	3.2	8.7	16.7
9	7.8	7.0	2.9	0.6	4.9	6.0
10	2.1	1.6	0.3	0.1	2.6	3.6
11	7.3	1.8	0.7	0.3	4.1	5.0
12	12.9	nd	0.7	nd	13.7	nd
13	nd	nd	nd	nd	nd	nd
14	0.8	4.2	0.1	0.1	4.3	7.6
15	0.8	0.6	0.1	0.2	1.9	1.0

Supplement 2a. Individual BALF data. Before and follow-up denote before infliximab treatment and at follow-up, respectively. Numbers are given as percentages, nd= not determined. Patient numbers refer to the same numbers as in Table 1.

Kullberg S, *et al. BMJ Open Resp Res* 2022; 8:e000933. doi: 10.1136/bmjresp-2021-000933

Patient	NK cells		CD56+ T cells		B cells	
	Before	Follow-up	Before	Follow-up	Before	Follow-up
1	18.9	28.8	7.4	12.9	15.7	3.0
2	16.9	11.6	16.2	14.1	3.6	3.0
3	15.6	14.8	11.9	11.3	13.3	10.3
4	32.5	31.1	11.8	11.6	3.5	3.0
5	2.5	nd	20.4	nd	1.2	nd
6	17.2	8.2	2.6	4.6	11.3	8.5
7	8.9	10.8	16.6	13.6	4.2	3.7
8	15.4	9.8	5.8	12.0	23.5	14.8
9	29.9	14.1	9.6	22.0	8.1	1.3
10	19.4	29.3	12.6	13.1	52.2	2.2
11	12.8	8.9	13.3	14.9	10.8	10.0
12	7.4	8.5	9.6	16.1	2.1	2.1
13	22.0	13.0	13.3	17.1	5.8	1.5
14	35.5	15.5	13.6	21.9	0.7	0.6
15	6.5	5.9	15.5	14.9	1.8	0.6

Supplement 2b. Individual PB data. Before and follow-up denote before infliximab treatment and at follow-up, respectively. Numbers are given as percentages, nd= not determined. Patient numbers refer to the same numbers as in Table 1.

Kullberg S, *et al. BMJ Open Resp Res* 2022; 8:e000933. doi: 10.1136/bmjresp-2021-000933

Patient	Cell concentration		Macrophages		Lymphocytes		CD4/CD8	
	Before	Follow-up	Before	Follow-up	Before	Follow-up	Before	Follow-up
1	153.7	74	91.5	62	5.5	7	12.4	1
2	201.5	153.2	89	94	9.6	5	3.6	4.1
3	507.2	306.8	72.2	70	20.5	23.7	7.6	4.4
4	232.2	nd	72.3	nd	16.3	nd	6.8	nd
5	153	nd	51.5	nd	47.7	nd	27.4	nd
6	142.2	265.1	59	38.5	38.1	60	12.6	4.1
7	135.5	125.3	79	88	20.3	11.3	10.9	1.4
8	175.6	283.9	75	73.3	22.3	22.6	12	1.5
9	116.6	41.3	72	80.8	23.8	17.5	7	3.4
10	320	288	62.7	46.6	17.7	8	12.1	4.4
11	177	338.7	88.5	77.7	10.3	21.2	4.3	2.3
12	358.6	nd	87.7	nd	8.6	nd	1.5	nd
13	nd	nd	nd	nd	nd	nd	nd	nd
14	170	304.8	67.8	41.8	27.4	57	7.1	10.2
15	601.9	443	83.5	68.5	10.5	14.7	7.4	14

Supplement 3. Individual data on cell concentration, macrophages, lymphocytes and CD4/CD8 ratio in BALF before infliximab treatment and at follow-up, respectively. Cell concentration is given as x10^6/l. Numbers for macrophages and lymphocytes are given as percentages, nd= not determined. Patient numbers refer to the same numbers as in Table 1.

Kullberg S, *et al. BMJ Open Resp Res* 2022; 8:e000933. doi: 10.1136/bmjresp-2021-000933
